# On the Use of Chloroform in Periodical Neuralgia

**Published:** 1849-11

**Authors:** Daniel Brainard

**Affiliations:** Prof. of Surgery in Rush Medical College


*On the use of Chloroform in Periodical Neuralgia.* By Daniel Brainard, M. D., Prof. of Surgery in Rush Medical College.—The districts where periodical fevers prevail, are also those where periodical neuralgias are most frequently observed; and these latter constitute a class of disease generally more obstinate and often more fatal than the febrile disease with which they are associated. Having often found them resist the action of quinine, arsenic, iron, narcotics, &c., I was induced, at length, to try the effect of the inhalation of chloroform, and with results so satisfactory that I am induced to offer the following cases, in illustration of its beneficial operation, to the profession.

I was called to visit Mrs. D., of Chicago; she was affected with a severe pain in the left eye, to which she had been subject, in the spring, for several years previously, and which has each time proved very severe and difficult of cure. The disease here existed a few days, occurring daily at 10 o'clock, A. M., occasionally attended with coldness of the extremities, great heat in the head, with excruciating pain in the eye, with profuse flow of tears and photophobia. The pain lasted in all its intensity about four hours, when it gradually diminished and passed off, its cessation being attended by a profuse perspiration.

The bowels being free, the patient was ordered sulph. quinine grs. x, pulv. opii grs. iij, at 6 o'clock, A. M., and be repeated at 9 o'clock, just before the expected return of the paroxysm. It returned with its usual severity, and the next day the same doses were repeated without any better results. At 12, A. M., of the same day, being called, on account of the intensity of the pain, I took a bottle of chloroform, and administered gradually by inhalation: the pain was greatly mitigated, and by frequent application was kept so light as to be easily borne. On the third day I was present at the hour of accession, and as soon as the symptoms of it appeared, used the chloroform to the effect of perfect insensibility. All symptoms immediately ceased, the patient fell into a sleep, with perspiration; and for upwards of a year, while under my observation, had no return of the disease, and enjoyed perfect health.

Case II. Mrs. A., residing ten miles north of Chicago was seized in December, 1848, with a severe pain in the left eye, extending to the forehead and temple, which was so severe as to cause delirium; with coldness of the extremities, and pulse scarcely perceptible at the wrist. The husband called for me in great alarm, but being unable to visit her that day, I gave him a prescription of sulph. quinine grs. v, pulv. opii gr. j, to be repeated every three hours till six doses had been taken.

On his return home, having been absent about three hours, he was much surprised to find his wife free from pain, and supposing all danger past, neglected to give the medicine until next day, when, at the same hour, the attack returned. I saw her about three hours after the attack and found her in a darkened room, with a bandage on her eyes, hands and feet cold, great heat in the head, and weak pulse. She had taken one dose of the medicine without any effect.

I immediately put a few drops of chloroform on a handkerchief, and requested her to inhale it, which she did with frequent smelling for thirty minutes, when she expressed some surprise that the pain was gone, and asked what it was she had been smelling of. In the course of a few minutes she raised the bandage from her eyes and conversed cheerfully for half an hour. I then directed her to take one of the powders morning and evening, and have since learned that she has had no return of the disease.

Case III. Miss N., a teacher, has been subject for several years to a neuralgia of the face, severe but irregular in in its situation and return. In March last she had an attack so severe as to unfit her for her ordinary pursuit. I prescribed sulph. quinine 5 gr. doses and directed her to inhale chloroform for the relief of the paroxysms. This she did but sparingly on account of a strong aversion to it, but found so great relief that she continued to employ it several times daily, in doses of about 20 drops, for two weeks, the attacks becoming gradually milder, and ceasing altogether at the end of that time.

This latter case is given to illustrate the difference between secondary neuralgia and that of a strictly periodic kind. In the former the chloroform seemed to have but a temporary effect, but increasing with frequent repetition, in the latter its influence is more decided and permanent, and corresponds to the influence of etherial inhalations, in paroxysm of regular ague, as described by Dr. Freer in a former No. of this Journal.—*Ib.*

